# Major limb amputations: A tertiary hospital experience in northwestern Tanzania

**DOI:** 10.1186/1749-799X-7-18

**Published:** 2012-05-11

**Authors:** Phillipo L Chalya, Joseph B Mabula, Ramesh M Dass, Isdori H Ngayomela, Alphonce B Chandika, Nkinda Mbelenge, Japhet M Gilyoma

**Affiliations:** 1 Department of Surgery, Catholic University of Health and Allied Sciences-Bugando, Mwanza, Tanzania; 2 Department of Orthopaedic, Catholic University of Health and Allied Sciences-Bugando, Mwanza, Tanzania

**Keywords:** Major limb amputation, Amputation patterns, Short-term outcome, Tanzania

## Abstract

**Background:**

Major limb amputation is reported to be a major but preventable public health problem that is associated with profound economic, social and psychological effects on the patient and family especially in developing countries where the prosthetic services are poor. The purpose of this study was to outline the patterns, indications and short term complications of major limb amputations and to compare our experience with that of other published data.

**Methods:**

This was a descriptive cross-sectional study that was conducted at Bugando Medical Centre between March 2008 and February 2010. All patients who underwent major limb amputation were, after informed consent for the study, enrolled into the study. Data were collected using a pre-tested, coded questionnaire and analyzed using SPSS version 11.5 computer software.

**Results:**

A total of 162 patients were entered into the study. Their ages ranged between 2–78 years (mean 28.30 ± 13.72 days). Males outnumbered females by a ratio of 2:1. The majority of patients (76.5%) had primary or no formal education. One hundred and twelve (69.1%) patients were unemployed. The most common indication for major limb amputation was diabetic foot complications in 41.9%, followed by trauma in 38.4% and vascular disease in 8.6% respectively. Lower limbs were involved in 86.4% of cases and upper limbs in 13.6% of cases giving a lower limb to upper limb ratio of 6.4:1 Below knee amputation was the most common procedure performed in 46.3%. There was no bilateral limb amputation. The most common additional procedures performed were wound debridement, secondary suture and skin grafting in 42.3%, 34.5% and 23.2% respectively. Two-stage operation was required in 45.4% of patients. Revision amputation rate was 29.6%. Post-operative complication rate was 33.3% and surgical site infection was the most common complication accounting for 21.0%. The mean length of hospital stay was 22.4 days and mortality rate was 16.7%.

**Conclusion:**

Complications of diabetic foot ulcers and trauma resulting from road traffic crashes were the most common indications for major limb amputation in our environment. The majority of these amputations are preventable by provision of health education, early presentation and appropriate management of the common indications.

## Background

Limb amputation is one of the most ancient of all surgical procedures with a history of more than 2500 years dating back to the time of Hippocrates [1,2]. Amputation has been practiced for punitive, ritual and therapeutic reasons including trauma, peripheral vascular disease, tumor, infection and congenital anomalies [3-5]. Limb amputation is considered the last resort when limb salvage is impossible or when the limb is dead or dying, viable but nonfunctional or endangering the patient's life [2].The loss of a limb by any individual, especially in developing countries where the prosthetic services are poor often has profound economic, social and psychological effects on the patient and their family [6-9] . Major limb amputations are essentially disfiguring operations that carry a fairly high perioperative mortality and morbidity and persons who have undergone amputations are often viewed as incomplete individuals [10].

The incidences of different pathologies leading to limb amputation have been reported to vary from one place to the other. In developed countries peripheral vascular disease ranks first as cause for amputation whereas trauma, infections, uncontrolled diabetes mellitus and malignancies are the leading cause for amputation in developing countries [11,12]. Most amputee patients in developed countries are older than 60 years of age, and 80-90% of lower limb amputations are performed as a result of vascular problems [10,13-15]. However in the developing world like Tanzania, the majority of amputees are young and the major cause of limb amputation varies from one hospital to another. Unfortunately, most often patients in developing countries presents late when limb salvage is not a viable option. This study was conducted to determine the pattern, indications and short-term outcome of major limb amputations in our setting and to compare our experience with that of other published information.

## Methods

### Study design

This was a cross-sectional descriptive study involving all patients who underwent major limb amputations at Bugando Medical Centre (BMC) between March 2008 and February 2010.

### Study setting

The study was conducted in the surgical and orthopaedic wards of Bugando Medical Centre (BMC), which is the Lake Zonal referral hospital situated in Mwanza city in northwestern Tanzania. It is a consultant and tertiary care hospital with a bed capacity of 1000. It is also a teaching hospital for the Catholic University of Health and Allied Sciences-Bugando (CUHAS-Bugando) and other paramedics. BMC provides services to patients from neighboring towns in Mwanza city and those referred from peripheral hospitals.

### Study population

The study population included all patients of all age group and gender who underwent major limb amputations at BMC within the period of study.

### Selection criteria

All patients of all age group and gender who underwent major limb amputation who consented for the study were included in the study. Patients who declined consent and those who were previously operated in other institutions, but required stump revision were excluded from the study.

### Recruitment of patients

Recruitment of patients was conducted after the decision to amputate the limb was made by the attending surgeon. The decision to amputate the limb, indications and levels of amputation was determined by the attending surgeon based on clinical evaluation, radiological investigations (e.g. plain x-rays of the affected limb, Doppler studies etc.) and histopathological investigations. Patients were screened for inclusion criteria and those who met the inclusion criteria were offered explanations about the study and requested to consent before being enrolled into the study. Amputation was performed by the attending surgeon who also prescribed the postoperative care of the patient. Major limb amputation was defined as any amputation at or proximal to wrist and ankle. In case where conversion to a higher level was required, the amputation level was recorded as the new revised level. Patients were followed up till discharge or death. Patients who developed complications were managed appropriately.

### Data collection and analysis

Data were collected using a pre-tested, coded questionnaire. Data included in the questionnaire were: demographic data (e.g. age, gender, education level and occupation status) and clinical data (e.g. indications, level of amputation, post-operative complications, Length of hospital stay mortality etc.). Data collected were analyzed using SPSS version 11.5 computer software and compared with the global literature on limb amputations to document our local trends and variations.

### Ethical consideration

The study was carried out after the approval by the department of surgery and BMC/CUHAS Ethics review board. All patients who met the inclusion criteria were requested to sign a written informed consent for the study.

## Results

A total of 162 patients underwent major limb amputations during the study period. The patients were aged 2–78 years (mean 42.30 ± 13.72 days). The majority of patients 156 (90.1%) were aged above 10 years of age. 108 patients (66.7%) were males and females were 54 (33.3%) with a male to female ratio of 2:1. The modal age group was 41–50 years (Table 1). The majority of patients (124; 76.5%) had primary or no formal education. One hundred and twelve (69.1%) patients were unemployed.

**Table 1:** Age group distribution

**Age group (years)**	**Number of patients**	**Percentage**
0-10	6	3.7
11-20	17	10.5
21-30	35	21.6
31-40	12	7.4
41-50	43	26.5
51-60	31	19.1
61-70	13	8.0
>70	5	3.1

Complication of diabetes mellitus (majority were Wagner’s classification stage 4 & 5) was the main indication for the major limb amputations in 68 (41.9%) patients followed by trauma in 62 (38.4%) patients and vascular disease in 14 (8.6%) patients respectively (Table 2).

**Table 2:** Indications for amputations

**Indication**	**Frequency**	**Percentage**
Diabetic foot	68	41.9
Trauma	62	38.4
RTAs	(37)	
Falls	(13)	
Assault	(10)	
Bite injuries	(2)	
Snake	1	
Crocodile	1	
Peripheral vascular disease	14	8.6
Malignancy	12	7.4
Squamous cell carcinoma	(6)	
Osteosarcoma	(4)	
Marjolin’s ulcer	(2)	
Post-burn contractures	3	1.9
Iatrogenic	2	1.2
Complication of POP	(1)	
Prolonged tourniquet use	(1)	
Congenital limb deformity	1	0.6

Trauma (mainly road traffic accidents and falls) and malignancies were most common indications for amputations in young adults (2^nd^ to 3^rd^ decades) whereas complications of diabetes and peripheral vascular diseases were the main indications in the 4^th^ to 5^th^ decades of age. Post-burn contractures, iatrogenic and congenital limb deformity were the most common indications in children aged 10 years and below (Table 3).

**Table 3:** Age group versus indications

**Age group**	**Diabetic foot**	**Trauma**	**PVD**	**Malignancy**	**PBC**	**Iatrogenic**	**Congenital limb deformity**	**Total**
0-10	-	-	-	-	3(1.9 %)	2(1.2 %)	1(0.6 %)	6(3.7)
11-20	-	14(8.6 %)	-	3(1.9 %)	-	-	-	17(10.5 %
21-30	-	31(19.1 %)	-	4(2.5 %)	-	-	-	35(21.6 %)
31-40	2(1.2 %)	10(6.2 %)	-	-	-	-	-	12(7.4 %)
41-50	36(22.2 %)	1(0.6 %)	3(1.9 %)	3(1.9 %)	-	-	-	43(26.5 %)
51-60	24(14.8 %)	3(1.9 %)	4(2.5 %)	-	-	-	-	31(19.1 %)
61-70	6(3.7 %)	2(1.2 %)	3(1.9 %)	2(1.2 %)	-	-	-	13(8.0 %)
>70	-	1(0.6 %)	4(2.5 %)	-	-	-	-	5(3.1 %)
Total	68(41.9 %)	62(38.4 %)	14(8.6)	12(7.4 %)	3(1.9 %)	2(1.2 %)	1(0.6 %)	162(100 %)

The major indications for upper limb amputations were trauma (42.3%) and malignancies (24.6%) while diabetic gangrene (45.5%) and trauma (32.2%) were the most common causes of amputation in lower limbs. The levels of amputation are given in Table 4.

**Table 4:** Levels of amputations

**Level of amputation**	**Frequency**	**Percentage**
Below knee amputation	75	46.3
Above knee amputation	64	39.5
Above elbow amputation	12	7.4
Below elbow amputation	10	6.2
Through knee	1	0.6
Total	162	100

Lower limbs were involved in 140 (86.4%) cases and upper limbs in 22 (13.6%) cases giving a lower limb to upper limb ratio of 6.4:1. There was no bilateral limb amputation. In the lower limb, the ratio of below knee amputation to above knee amputation was 1.2:1 and in the upper limb, the ratio of below elbow to above elbow was 1.2: 1. A total of 46 patients (28.4%) required additional procedures. The most common additional procedures performed were wound debridement, secondary suture and skin grafting in 42.3%, 34.5% and 23.2% respectively. Two-stage operation (e.g. initial guillotine amputation and later stump revision or change of amputation level from below to above) was required in 45.4% of patients. Post-operative complications occurred in 54 patients (33.3%). Details of post-operative complications are shown in Table 5.

**Table 5:** Post-operative complications

**Complications**	**Frequency**	**Percentage**
Surgical site infection	34	21.0
Revision amputation	16	9.9
Phantom pain	9	5.6
Wound hematoma	7	4.3
Wound dehiscence	5	3.1
Stump gangrene	3	1.9

Surgical site infection (SSI) was the most common post-operative complication accounting for 21.0% of cases. Of these, 24 (70.6%) specimens had positive bacterial growth within 48 hours of incubation while 10 (29.4%) had negative bacterial growth. Four out of 24 cultured specimens (16.7%) had polymicrobial bacterial growth while 20 (83.3%) had pure bacterial growth. *Staphylococcus aureus* was the most common bacteria isolated (7; 29.2%), followed by *Escherichia coli* 5 (20.8%) and *Klebsiella pneumoniae (*4; 16.7%) *Pseudomonas spp* and *Proteus spp* were the least bacteria isolated. Anaerobic cultures were not done.

Hospital stay of patients ranged from 9 to 58 days with the mean duration of 22.4 days. The majority of patients (108: 66.7%) had good recovery. Two (1.1%) left against medical advice and one (0.6%) patient absconded. There were a total of 27 deaths giving a mortality rate of 16.7%. The main causes of deaths were complications of diabetes in 42.8% of cases, wound sepsis in 40.5%, advanced malignancy with metastasis in 4.7% and not established in 2.8% of cases.

## Discussion

Since it was first described by Hippocrates in 460–377 BC, limb amputation has been a common surgical procedure performed by orthopedic, general, vascular and trauma surgeons for therapeutic reasons to serve patient’s life. However, it is often associated with profound economic, social and psychological effects on patient and their family [1,2]. As amputation indications and patterns vary between hospitals in a country and between countries, this study was undertaken to describe our experiences on major limb amputations in a larger tertiary care teaching hospital and compare the findings with similar studies conducted in other parts of the world with a view to highlighting the variations in the pattern and indications for amputations. This would enable meaningful preventive measures to be proffered.

The male preponderance among amputee in the present study agrees with the findings by other authors [6,7,16-18]. We could not find any reasons to explain for the male preponderance in this series.

The majority of our patients were in the 4^th^ and 5^th^ decades which is comparable with other studies [6,10,18,19] but in contrast with another study in Ghana which reported high peak age incidence in the 7^th^ decade [20]. Other studies reported even lower peak age incidence [3,21].

This age differences can be explained by variation in the cause and patterns of amputation which tend to vary between hospitals in the country and between countries.

Complications of diabetic foot ulcers were the most common indication for major limb amputation in our study, followed by trauma and peripheral vascular diseases. Similar trend was also reported in other series [10,20-23]. A similar pattern was also seen in the West where Pohjolainen & Alaranta [24] reported that 49% of amputations in Finland resulted from diabetic complication. These findings are not in agreement with other studies which reported trauma as the most common indication for major limb amputation [2,6,18,25]. These differences in the pattern of indications reflect differences in incidences of different pathologies leading to limb amputation which tend to vary from one place to the other. The increased incidence of diabetic foot complications requiring lower limb amputation may reflect the level of effectiveness of the early detection of diabetes mellitus and the foot at risk, medical education, patient compliance and overall control of diabetes mellitus in this population. The risk of amputation in diabetic patients is increased up to 15 fold [26]. Factors contributing to this include sensory, motor neuropathy causing gait abnormality and deformity; autonomic neuropathy causing abnormal blood flow; macrovascular disease causing ischemia; poor glycaemic control causing increased risk of infection. Inadequate care of infection and ulceration is also a potentiating factor for limb amputation [10,26].

In the present study, although trauma (commonly due to road traffic crashes) ranked second, it was found to be the most common indication for amputation in young adults in their productive and reproductive age group. Limb amputation in this group almost always result in a serious economic crisis for the family, especially that prosthesis are either unavailable or unavoidable [27,28].

Studies have shown that approximately 80-90% of limb amputations in developed countries are performed as a result of vascular problems [13-15].

In our study, peripheral vascular diseases ranked third as indication for major limb amputation. This may be due to complications of diabetes mellitus. However, peripheral vascular disease (PVD) unrelated to diabetes mellitus was the most common cause of lower limb amputations in Kenya, contrary to the belief that PVD is only common in developed countries [22]. Further study is needed to explain such observation in our environment.

Postburn contractures commonly involving the upper limbs were the most common indications for amputation in children less than 10 years of age. This was followed by iatrogenic indications (prolonged use of tourniquet & complication of POP).and congenital limb deformity. Paudel *et al.*[2] in Nigeria reported post-burn contractures as the most common indication for amputation in children followed by congenital limb deformity and tumors. Increased incidence of post-burn contractures as indication for limb amputation reflects poor management of acute childhood burn injuries in these two populations.

In agreement with other studies [3,6] most of our amputations were performed in the lower limbs and below knee amputation was the most common procedure performed. This finding confirms the earlier findings that lower extremities are injured more often than the upper extremities and diabetic gangrene is common on the lower extremities than elsewhere on the body [5,29,30]. However, other studies reported above knee amputation as the most common procedure performed than below knee amputation [3,18,31].

Late presentation with spreading gangrene or advanced diabetic foot gangrene or malignant lesions that have involved the underlying bones may force the surgeon to opt for a higher level of amputation [20,27,28].

The complication rate (33.3%) in our study is lower compared with that of Essoh *et al.*[6] in C↖Ðte d’Ivoire (39.0%). Surgical site infection was the most common complication in the present study and *Staphylococcus aureus* was the most common organism isolated. Similar microbiological trend was also reported by other authors [6,18]. The overall surgical site infection rates in these studies reflect the severity of complications leading to amputation. Indeed, the majority of patients in these studies presented with spreading gangrene or diabetic septic foot.

The rate of re-amputation in our patients (9.9%) was found to be lower compared to that reported by Essoh et al. [6] in C↖te d’Ivoire (23%) but higher than that reported by Kidmas *et al.*[18] in Nigeria (7.4%). These differences in re-amputation rates may be explained as follows; firstly, late presentation with advanced disease increases the risk of revision amputation. Secondarily, the majority of amputations are performed by junior doctors with little experiences. Thirdly, high complication rates in our patients may also increase the risk of re-amputation. Fourthly, poor management of amputation stump is also a risk factor for re-amputation.

The mean duration of hospital stay in our study was 22.4 days, which is high than that reported in other studies [10,32], but lower than that reported by other authors [6,33,34]. The length of hospital stay is an important measure of morbidity. Estimates of length of hospital stay are important for financial reasons, and accurate early estimates facilitate better financial planning by the payers. The duration of hospital stay has been identified as one of the main determinants of cost associated with amputation [35].

The mortality rate in the present study (16.7%) is comparable with that reported in other studies [6,7,18,23], but significantly higher than that reported by Massod et al. [10] in Pakistan. The reasons for high mortality rate in our study are diabetic-related complications, wound sepsis and advanced malignancies with metastasis which were found to be common in our study.

## Conclusion

Complications of diabetic foot ulcers and trauma resulting from road traffic crashes were the most common indications for major limb amputation in our environment. The majority of these indications are potentially preventable through provision of health education, early presentations and adequate treatment of these conditions. Good diabetic control and early recognition and management of risk factors for foot complications, measures on prevention of road traffic crashes and community health education to encourage early presentation to hospital will reduce the number of patients undergoing major limb amputations in this region and subsequently reduce the number of amputee.

## Competing interests

The authors declare that they have no competing interests.

## Authors’ contributions

PLC - Study design, data analysis, manuscript writing, editing and submission of the manuscript, JBM, RMD, IHN, ABC and NM participated in data analysis, manuscript writing & editing and JMG- coordinated and supervised the manuscript writing & editing. All the authors read and approved the final manuscript.
